# IPA Improves Muscle Function and Reduces Bone Loss in Aged Mice and Modulate Lifespan in Drosophila melanogaster

**DOI:** 10.1093/geroni/igaf122.3971

**Published:** 2025-12-31

**Authors:** Sagar Vyavahare, Ford Berger, Shelton Swint, Roger Zhong, Marion Cooley, Carlos Isales, Jessica Hoffman, Sadanand Fulzele

**Affiliations:** Augusta University, Martinez, Georgia, United States; Augusta University, Augusta, Georgia, United States; College of Science and Mathematics, Augusta, Georgia, United States; Augusta University, Augusta, Georgia, United States; Dental College of Georgia, Augusta, Georgia, United States; Augusta Universoty, Augusta, Georgia, United States; Augusta University, Augusta, Georgia, United States; Augusta University, Augusta, Georgia, United States

## Abstract

Aging is associated with alterations in endogenous tryptophan (TRP) metabolism that contribute to musculoskeletal decline. In this study, we investigated the effects of the microbiota-derived tryptophan (TRP) metabolite, indole-3-propionic acid (IPA), on musculoskeletal health in aged mice. Additionally, we assessed the impact of IPA on the lifespan of the fruit fly, Drosophila melanogaster. Our findings revealed that IPA-treated aged mice exhibited enhanced muscle function (grip strength and hang time). Histological and bone microCT analyses revealed no changes in muscle fiber size but enhanced bone microarchitecture. Furthermore, molecular studies have elucidated that IPA treatment prevents oxidative stress and reduces senescence, indicating improved cellular survival. Our Drosophila melanogaster longevity analysis revealed a significant extension of lifespan, but lifespan effects were genotype- and sex-specific. Collectively, our findings identify IPA as a promising microbiota-derived metabolite that improves musculoskeletal health and promotes longevity, highlighting its potential as a therapeutic intervention for age-related decline.

